# Round the Hospitals

**Published:** 1920-07-24

**Authors:** 


### ROUND THE HOSPITALS.

The *Daily Telegraph* Appeal for Nurses is now closed, and the correspondence between Sir Arthur Stanley and Lord Burnham, published in the *Daily Telegraph* of July 19, confirms and amplifies the forecast made at the general meeting of the College of Nursing that the Tribute Fund of the Nation's Fund for Nurses had nearly reached the sum of £100,000. Over a quarter of a million shillings, or, to be exact, £13,883 12s., has been subscribed through the columns of the *Daily Telegraph* in response to appeals made by that paper and by the *Leicester Mail*. All expenses of collection have been defrayed by the proprietors. The indirect consequences of Lord Burnham's support of the Nation's Fund for Nurses have been even more valuable. Nurses all over the world have benefited

**ROUND THE HOSPITALS**—(*continued*).

from the able manner in which their aims for the future and the needs of their suffering sisters have been put before the public in this influential organ There is perhaps no greater power than that which is wielded by public-spirited newspapers, unswayed by personal ambitions, and to have the *Daily Telegraph* wholeheartedly on its side is a definite achievement, which marks a distinct stage in the evolution of the College of Nursing. We are fully persuaded that the current of interest in nursing ideals thus set stirring among readers of the *Daily Telegraph* will not be suffered to stagnate, and we venture in the name of our own readers to thank Lord Burnham and his coadjutors for all they have done for nurses.

The members of the College of Nursing will not be surprised to find that Sir Arthur Stanley's letter to them on the subject of the registration fee has greatly excited the opposition. The frank offer to pay the fee under certain conditions for members of the College who believed that whatever Registration Bill became law they would automatically, without further fee, be placed on the State Register, has so completely vindicated the College that there is nothing further to be said. The original leaflet issued in 1916 definitely stated that if and when the "Nurses' Registration Bill" **drafted by the Council of the College** became law members of the College would "automatically and without further fee be placed upon the State Register," for the College Bill provided that the Register already formed by the College of Nursing should be the first Register under the Act. The following is the whole of the paragraph in question contained in the leaflet:

"1. Because the Council of the College of Nursing has drafted a 'Nurses' Registration Bill' which provides that the Register already formed by the College of Nursing shall be the first Register under the Act. If, therefore, you are on the College Register you will automatically and without further fee be placed upon the State Register when the 'Nurses' Registration Bill' is passed."

We notice that the *British Journal of Nursing*, in its current issue, quotes only part of the paragraph and omits the quotation marks. The inference is plain.

It is pleasant to learn that Sir Arthur Stanley's letter has produced a large number of letters from members expressing appreciation of and gratitude for what the College has done for nurses. Many send a guinea for the State Registration fee, but the secretary points out that until the regulations now being drafted by the General Nursing Council are published the amount of the fee remains unknown; it cannot be more, but it may be less than one guinea. Members of the College, and all trained nurses, should watch the Press for the appearance of these regulations and then carry out the instructions of the General Nursing Council. They will, no doubt, be required to apply individually to the Registrar of the General Nursing Council for application forms, and to pay their fees direct. Then if any College member desires the College to pay the State Registration fee under the conditions named by Sir Arthur Stanley she should write to the secretary of the College enclosing the State Registration receipt. Very few members up to the present desire to take advantage of this offer, realising the necessity to support the College in the carrying out fully of its programme of advancement for the whole profession.

The interesting lectures on public speaking given early this year by Miss Bagley and Mrs. Hicks, under the auspices of the London Centre of the College of Nursing, gave evidence of so keen a desire for knowledge on the subject that the London Centre is arranging for a course of lessons in voice-training. This is to be given by Miss Umfreville Hicks, of the Polytechnic School of Speech Training, at 7 Henrietta Street, Cavendish Square, W. 1, and to begin in October. The fee for a class of twelve persons for ten lessons will be £5 5s. The classes will be given once a week, on a Tuesday or Thursday evening from 6.30 to 7.30. Miss Bompas, London Centre Club, 7 Henrietta Street, W. 1, will be glad to hear not later than August 16 from members wishing to join, and asks them to mention which evening will be most convenient. Providing a class of twelve can be arranged the cost to each member would be the small sum of 8s. 9d. for the whole course.

H.R.H. Princess Louise Duchess of Argyll paid a visit on July 15 to the Maternity Charity and District Nurses' Home, Plaistow. She was received by Miss Davies, the Lady Superintendent, Dr. Kennedy, the Medical Officer, and Mr. Andrews, the Secretary, whilst the superintendents of the various departments of the Central Home and its branches formed a guard of honour. H.R.H. spent some time in the grounds of the Institution, where she gave an address to about 200 pupils, and she then inspected the home, the maternity and child welfare centre, the milk depôt, and the hospital. Her inspection appeared to give her great pleasure, and at the conclusion of her visit H.R.H. congratulated the nurses on their work. Before leaving H.R.H. visited the oratory attached to the home.

The anxiously expected report of Mr. Frederick Acton on the management of the Newark Hospital has been issued, and, as was confidently expected from the able manner in which the inquiry was conducted, is an impartial and useful document. Mr. Acton finds that, having regard to the fact that the hospital was exceptionally full and the nursing staff admittedly overworked during Mr. Lewis Ransome's stay in the private ward, in the main he was well cared for. Mrs. Ransome's frequent visits to the general wards and her interference with the patients and nurses are alluded to with regret, as also the tone adopted by the matron in consequence. The position of an overworked matron labouring with an inadequate staff to deal with an unusual number of patients, and confronted by the continual presence of an exceedingly anxious wife whose husband is lying betw[illegible]

**ROUND THE HOSPITALS—(*continued*).**

life and death, has in it all the elements of friction, and Mr. Acton seems to have done his best to put himself in the place of both ladies. The engagement of a special nurse for Mr. Ransome, as suggested in this report, was the proper course to pursue. Other incidents reflecting on the quality of the nursing and general discipline are dealt with in the report in a judicious spirit. Mr. Acton recommends the addition of another trained nurse on the staff, preferably with a knowledge of massage, of a qualified dispenser, and also perhaps of another wardmaid. He also draws attention to the need for some small sub-committee meeting weekly to which the matron and house surgeon could report.

---

The Newark Hospital difficulties open up a matter of general interest to the public at the present moment when a general extension of schemes for pay-wards in hospitals and even in Poor-law infirmaries is taking place. Mr. Acton discovered in the course of his inquiry into Mr. Ransome's complaints that there are risks in admitting paying patients to hospital, that "unless you can separate the care and treatment from the ordinary cases there may be overlapping and favouritism as regards the nursing." The laxity of rule in such cases as to visitors as well as the variation in diet tends to dissatisfaction. There is a "temptation to nurses to favour such paying patients," or, at any rate, "a suspicion that they do so at the expense of the ordinary patient, for whom alone, according to the present rules, the hospital is intended." It is very desirable that these dangers should be in the minds of managers, and that care should be taken to avoid them. In larger hospitals the pay-wards are usually quite distinct, but in smaller ones paying patients often occupy beds side by side with ordinary patients, and the result may be very unsatisfactory.

---

The Merthyr Union Infirmary in South Wales appears to be badly in need of reorganisation. One of the guardians is reported as saying: "We want a re-shuffling of the staff in this institution, and if every one is cleared out it will be to the advantage of its administration." If this is a specimen of the manner in which the guardians regard their duties to their staff we are not surprised that there have been many resignations among the trained nurses. It would seem that they have been attempting to run the institution on too small a staff, and the expedient of withdrawing from the probationers their weekly eight hours' leave on account of the shortage of nurses has proved as unsuccessful as it deserved to be. The privilege has been restored and an additional nurse is to be engaged. We suggest that they take their superintendent nurse into their confidence, accept her recommendations, and strengthen her powers. It is satisfactory to know that a Committee of eight members has been appointed to investigate the affairs of the infirmary.

At an enthusiastic meeting held on July 10 at the Chelsea Hospital for Women the Chairman, Sir F. W. R. Fryer, stated that there was a balance of £7,500 over from the building fund towards the new nurses' home, which it is estimated will cost from £18,000 to £20,000. At present the nurses are housed in the hospital, an arrangement which always has its drawbacks. When they are accommodated in detached quarters fifty additional patients can be received, so there is every motive for pushing forward the scheme. Next year the Chelsea Hospital for Women celebrates its jubilee and is anticipating that the occasion will bring grist to the mill. General Sir Francis Lloyd, enforcing the appeal for the nurses' home, paid a tribute to the splendid work performed by nurses during the war.

---

A group of nurses is to take part in the procession, emblematic of the League of Nations, which is to take place on the August Bank Holiday round Hampstead Heath to celebrate the anniversary of Peace Day, July 19, 1919. The details of the procession were decided on at a meeting held at Lord Leverhulme's house in Hampstead on July 17. Various cars will be fitted up to represent the ideas of war and peace. Nurses will accompany the one containing the wounded, and to our mind they should also be invited to take part in the presentations of peace. It is likely that for generations to come they will be busily occupied in the task of assisting the nations to replace cruelty and disease by loving-kindness and methods of hygiene. All who want to help are invited to communicate with Mrs. S. A. Barnett, C.B.E., 1 South Square, Hampstead Garden Suburb.

---

Dame Maud McCarthy and Dame Ethel Becher, with Miss Cox Davies, sat in council with the Dowager Countess of Airlie and others at an At Home given last week at 3 Grosvenor Place, to discuss the prospects of the proposed "United Nurses' Services Club" which we mentioned lately. Lady Airlie spoke with confidence of Queen Alexandra's sympathetic support, and Sir Alfred Keogh and Sir Anthony Bowlby spoke warmly in favour of the proposal. Miss Cox Davies mentioned that it is hoped to start the Club on the lines of a first-class residential ladies' club, with about twenty bedrooms. The essential thing is the requisite financial support, and recent experience in ladies' clubs, which are booming in the existing shortage of accommodation for visitors to London, seems to show that a large number of members can be counted on for support to the extent of, say, £25 to £50, if any really sound financial scheme is formed which promises security to their modest outlay of capital.

---

The new pensions warrant dated July 2 issued by the War Office is one of great moment to nurses disabled in the war. It is published as a Command paper (Command 811) and a special Army Order, and can be obtained from H.M. Stationery Office.

**ROUND THE HOSPITALS—*(continued)*.**

For nursing sisters the temporary bonus will from now on be added to the pension, and in some cases a further increase has been made. Matrons totally disabled will now receive £180 a year instead of £125; sisters and staff nurses £150 instead of £100. The maximum addition to service pension is raised from £75 to £90. All these alterations will be made without application for them. But in one class of case special application must be made within one year from the date of the warrant to the Special Grants Committee, S.G.E., Millbank House, Millbank, S.W. 1. This provision applies to disabled nurses whose pre-war earnings exceeded £95 a year. These pensioners may claim an alternative pension up to a maximum of £250 a year, the sixty per cent. addition to pre-war earnings being allowed.

---

H.M. The King has been pleased to confer on Miss Geraldine Mathews, R.R.C., Matron of the National Children's Hospital, Dublin, the Medal of the Order of the British Empire "for courage and devotion in rendering aid to wounded under fire." The *Dublin Daily Express* records that Miss Mathews' personal efforts and conspicuous bravery were the means of saving three valuable lives.

---

Miss Winifred Todd, of the Rotunda Hospital, Dublin, has been appointed out of five selected candidates to be matron of the new Wellhouse Hospital under the Barnet Board of Guardians. Everyone is delighted, because she is a member of an old Barnet family, and lived there for fifteen years. She took a term of preliminary training as a girl in the Victoria Cottage Hospital, Barnet, and has had a successful nursing career, including service in more than one military hospital during the war. The present superintendent nurse of the Barnet Infirmary has been appointed assistant matron at a salary of £100 a year at the Wellhouse Hospital, which is to be formally opened shortly with what the Chairman described as a "big splash."

---

*The Times* announces that Miss Sinclair, matron of Kingston Union Infirmary, has sent in her resignation, following on the Guardians' request at their meeting as reported in our last week's issue, p. 417.

---

The nurses at the Macclesfield Infirmary sent a deputation to the Guardians a month ago asking the Board to make them a ration allowance while on holiday. The male staff of the workhouse have long enjoyed this privilege, and it was believed that the Guardians would see the justice of equal treatment being meted out to the nurses. Moreover, the Chairman of the Board, the House Committee and the Hospital Committee have gone carefully into the matter, and are unanimously in favour of making the concession. Notwithstanding this influential backing, the Board not only refused by a majority to grant the ration allowance, but have actually declined to allow the nurses to bring their very strong case again before them. It is very usual, if not yet quite universal, for the rations allowance to be made, and the parsimony exhibited by the Macclesfield Guardians is the worst possible policy, to put it on the lowest ground, in these days when good nurses are hard to get and very easy to lose. The Board might easily have to spend as much on advertising as would pay the nurse's board twice over in their holidays. We should much like to know what salaries they are paying to their staff.

---

A meeting has been called for July 28 at the Queen Mary Hostel, 194 Queen's Gate, to consider by what means this much-valued institution can be placed on a permanent basis for the accommodation of military and civil nurses making a short stay in the Metropolis. At present the requests for beds from nursing sisters passing to and from the Armies of Occupation, and indeed, all parts of the world are so numerous that to close it down would be a real calamity. Members of the naval and military nursing services, the Ministry of Pensions Service, the Queen Victoria Jubilee Institute, the Overseas Nursing Association, and the Red Cross Nursing Services of various countries are eligible for admission, and up to last September the number admitted totalled nearly 24,000. The Australian Red Cross gave a tablet to the hostel to commemorate the hospitality afforded to Australian nurses, and this was fixed in position not long ago by the Queen herself. No charge even for laundry is made to the guests.

---

The Queen of Spain, whose presence in town is attended by many demonstrations of popular interest, visited the League of Remembrance Depôt at Marlborough Gate, in company with Miss A. S. Beatrice on the 13th inst. Her Majesty was picturesquely attired in a mauve satin charmeuse gown, a black hat with plumes, and a double chain of pearls. The Royal party were shown over the Depôt by Mrs. G. H. Gibson, and a bouquet of mauve and pink carnations was presented by Miss Aileen Fleming. The Queen specially admired the babies' garments in course of making for Infant welfare centres and the admirably fitted department for preparing surgical dressings.

---

We note an excellent article, of the kind to do real good, on the subject of "Educated Women as Nurses" in a recent issue of the Educational Supplement of *The Times*. The writer was present at the conference on attracting women to the profession held by the College of Nursing, and warmly supports the case for a higher standard of education in entrants. In particular, the need for women of education to undertake the preventive side of mental nursing is brought into prominence.

